# Editorial: The Potential of School-Based Interventions That Target Executive Function

**DOI:** 10.3389/fpsyg.2022.831745

**Published:** 2022-01-27

**Authors:** Robin Jacob, Tyler W. Watts, Antje von Suchodoletz

**Affiliations:** ^1^Institute for Social Research, University of Michigan, Ann Arbor, MI, United States; ^2^Teachers College, Columbia University, New York, NY, United States; ^3^Department of Psychology, Science Division, New York University Abu Dhabi, Abu Dhabi, United Arab Emirates

**Keywords:** intervention programs, executive functions, academic performance, schools, variation

## Introduction

The impetus for this special issue was a paper published several years ago by one of the guest editors, which explored the question of whether or not executive function (EF) skills were causally related to children's academic achievement (Jacob and Parkinson, 2015). The conclusion of the paper, which reviewed 67 studies regarding the relations between EF and achievement, was that much more research was needed. Although numerous correlational studies had shown links between EF and academic achievement, the existing research provided little rigorous evidence that the relationship was causal.

Since the publication of the original paper, the study of EF and schooling has advanced. As Mattera et al. summarize in their paper in this special issue, there have been more rigorous studies of school-based interventions that target EF, particularly in the preschool setting. Results have yielded mixed findings. Some studies, particularly of behavioral-focused interventions, have found impacts on EF skills. A few have demonstrated impacts on academic outcomes. But there is enough variation in intervention type, measurement, and implementation context to make definitive conclusions difficult. The authors highlight four issues that still need further study: intervention timing, contextual factors that drive effects, the relationship between EF and other outcomes, and the role of scale in improving EF. The remaining papers in this Research Topic help shed light on some of these questions.

## Intervention Timing

Gunzenhauser and Nückles explore the influence of intervention timing. They suggest that early intervention may have the greatest potential because behavioral and cognitive demands of the school setting are new during early childhood, and EF skills are especially needed to help children successfully adapt. This suggestion is in line with the approach that most school-based interventions have taken. From a developmental perspective, intervening earlier seems an obvious choice, as these skills may be most malleable in early childhood. Yet, this line of argument also suggests that the impacts of EF training on academic outcomes would be greatest in the short-term. Yet several studies have found evidence that early EF skills predict *later* academic skills (e.g., Duncan et al., 2007; Caroline and Pagani, 2012), and others have hypothesized that boosting early EF skills might lead to lasting impacts on long-term outcomes (e.g., Deer et al., 2020). Further, as Mattera et al. note, several EF training programs find impacts on both EF and achievement in early elementary school. We currently know little about how interventions later in the developmental period compare to those that intervene earlier.

Kim et al. paper suggests that early schooling by itself may not have a strong impact on the development of children's EF skills. This might imply, as Gunzenhauser and Nückles recommend, that more specific and targeted approaches to developing EF skills are needed.

## Which Intervention Factors Drive Effects?

Mattera et al. hypothesize that a curricular focus or integration with cognitive skills may be needed to maximize the impact of school-based EF interventions. Gunzenhauser and Nückles similarly hypothesize that for EF interventions to be effective in far transfer tasks like academic achievement, they may need to be targeted to a specific area of academic growth.

Others in this special issue focus on questions related to implementation. Barnes et al. submit that short, targeted, easy to implement interventions that require limited training (in this case *Brain Games* that teachers can use on an *ad hoc* basis) may have the potential to improve EF and self-regulation outcomes. Their study, although far from definitive, shows some promise that *Brain Games* are effective. Goble et al. also hypothesize that implementation is an important factor driving outcomes. They explore the implementation of the *Tools of the Mind* program, and find some evidence that teachers who implement with higher fidelity yielded higher gains in child EF skills. They also find that fidelity was moderated by a variety of teacher characteristics, most importantly, years of experience. Less experienced teachers both implemented with higher fidelity and had children with higher EF gains, perhaps because less experienced teachers are more malleable in their teaching approach. Finally, Shea and Jenkins highlight the importance of exploring heterogeneity of effects, noting that a simple focus on mean outcomes may miss important impacts.

## Final Thoughts

The articles in this issue all pose interesting hypotheses about the potential effectiveness of school-based programs designed to impact EF and/or achievement. Yet, more research is still desperately needed. All of the theories and programs explored in this volume need further study. Only a handful of studies have examined the far transfer effects of pure EF interventions implemented in schools. Many existing studies combine academic interventions, broader social-emotional interventions, and executive functioning interventions, making it difficult to tease out the operative factors. The overlapping foci of these studies hamper our ability to make progress on crucial conceptual issues, and they can blur the definition of EF. We need studies with internally valid causal designs that can isolate specific skills that fall under the larger EF umbrella to help us better understand how specific aspects of EF relate to one another and to academic achievement in the short- and long-run. It would, for example, be interesting to directly test the proposition that focusing on a very narrow type of EF training specific to an underlying academic skill could yield positive far transfer effects, as Gunzenhauser and Nückles suggest. Similarly, it would be illuminating to test whether an EF intervention implemented in elementary/middle school leads to better academic outcomes than one implemented in preschool, in both the short- and long-term. Finally, as noted by several authors in this volume, both definitional and measurement issues continue to confound intervention studies. It will be difficult to come to definitive conclusions about intervention effectiveness without more clearly defined constructs and a consistent set of measures. We hope this special issue will spur continued research in this area so that we can gain a better understanding of the potential for school-based EF interventions to yield lasting impacts for children.

## Author Contributions

RJ, TW, and AS contributed to the conceptualization of the editorial. RJ wrote the manuscript. TW and AS edited the manuscript. All authors contributed to the article and approved the submitted version.

## Conflict of Interest

The authors declare that the research was conducted in the absence of any commercial or financial relationships that could be construed as a potential conflict of interest.

## Publisher's Note

All claims expressed in this article are solely those of the authors and do not necessarily represent those of their affiliated organizations, or those of the publisher, the editors and the reviewers. Any product that may be evaluated in this article, or claim that may be made by its manufacturer, is not guaranteed or endorsed by the publisher.
